# Editorial: Growing up and moving forward: Discontinuing Primer Notes

**DOI:** 10.1002/aps3.11293

**Published:** 2019-10-08

**Authors:** Theresa M. Culley

**Affiliations:** ^1^ Department of Biological Sciences University of Cincinnati 614 Rieveschl Hall Cincinnati Ohio 45221‐0006 USA

As a resource for innovative methods and techniques, *Applications in Plant Sciences* (*APPS*) has expanded and diversified since it was first launched. Now in its seventh year as an independent publication of the Botanical Society of America, the journal has grown to better serve the plant sciences community—from publishing exclusively genetic primer notes to featuring new protocols, cutting‐edge tools and software, methods reviews, applications of new techniques, and genomic resources. As such, our articles now span the entirety of the plant sciences, including ecology, genetics, physiology, development, bioinformatics, and systematics. To focus on these broader areas, our editorial team has determined it is time for an important change in the direction of *APPS*.

Beginning 31 October 2019, *APPS* will discontinue the Primer Notes category and will no longer accept submissions for articles solely reporting newly developed genetic primers. This new policy recognizes certain realities. First and most important, the amount of time and effort necessary to generate primers, particularly microsatellite markers, has greatly diminished in recent years due to the declining cost of technology such as next‐generation sequencing; primers that once took months to painstakingly develop from bacterial libraries, and cost thousands of US dollars if outsourced, can now be readily identified in most research laboratories in only a few days with minimal cost. Consequently, it is now more appropriate to include primer development as one of several methodological steps within a larger study, rather than for these markers to be published as individual reports.

In addition, we have increasingly found that the Primer Notes category has not always been used as originally intended. We have seen an increase in published primers that are never subsequently used or cited (including by the authors who originally developed the primers), even accounting for rarity of the focal species. This does not align with the original objective of the Primer Notes category in *APPS* as a repository for valuable markers of use to the broader plant sciences community.

Incorporating primer development as part of a larger research study is the way of the future. In fact, inclusion of primer development is now almost standard in single‐length nucleotide polymorphism (SNP) studies (e.g., Cordeiro et al., 2019) and is becoming increasingly common for papers employing microsatellite markers (e.g., Tsy et al., 2013; Marques et al., 2015; Thompson et al., 2015; Stokes et al., 2019; Zhang et al., 2019). In these cases, primer development is either integrated directly into the methods (positioned between the DNA extraction and PCR steps) or placed within an appendix or data supplement and referred to within the main text. Because the inclusion of primer development within a full study is still a relatively recent development, some authors may need to inquire with a journal's editorial office about their policy of how to best incorporate primer development before submitting their manuscript. Ultimately though, we expect this process to become standard as more researchers and journals understand the role of primers as being part of larger studies.

Retiring Primer Notes will allow *APPS* to focus its resources more strategically on other article types that are growing in popularity and usefulness, including Protocol Notes, Software Notes, and Review Articles. *APPS* has also been particularly successful with recent special issues addressing new methods and applications in areas such as phenological research (March 2019; Ellwood et al., 2019) and belowground botany (April 2019; Pec et al., 2019). Upcoming special issues planned for 2020 will focus on plant phenomics, low‐cost methods in the plant sciences, and machine learning in plant biology. By exploring innovative areas like these, we will ensure that *APPS* continues to grow as a broad and valuable resource for plant biologists in a rapidly changing technological landscape.
